# 

**Published:** 1883-11

**Authors:** 


### CONTENTS.

### COMMUNICATIONS.

The Conservative Treatment of the Dental Pulp when Exposed, versus Devitalization ........ 513
Replantation of Teeth ........ 525
Clinics ........ 528
Amalgam as a Material for Filling Teeth ........ 533
Sandy Foundations ........ 540
A Case of Plastic Surgery ........ 543
Nomenclature ........ 546

### SELECTIONS.

Æsthetic Dentistry ........ 548
Article on Dentition ........ 553
Metastatic Choroiditis After the Extraction of a Molar Tooth ........ 555
Breakfast Beverages ........ 556
A Peculiar Fracture of the Lower Jaw ........ 557
The Development of Cancer from Non-Malignant Diseases ........ 558
Notched Teeth ........ 558
Trichlorophenate of Lime ........ 559
Resorcin as a Dressing ........ 560
Inodorous Iodoform ........ 560
Health Aphorisms ........ 560
How Shall we Blister? ........ 561
Proceedings ........ 561

### EDITORIAL.

Care of Teeth ........ 562
Obituary ........ 564

